# Diagnostic Performance of the QIAstat‐Dx Meningitis/Encephalitis Panel: Insights From Seven Clinical Evaluations

**DOI:** 10.1002/mbo3.70079

**Published:** 2026-02-20

**Authors:** Flora Marzia Liotti, Brunella Posteraro, Maurizio Sanguinetti, Giulia De Angelis

**Affiliations:** ^1^ Dipartimento di Scienze di Laboratorio ed Ematologiche Fondazione Policlinico Universitario A. Gemelli IRCCS Rome Italy; ^2^ Dipartimento di Scienze Biotecnologiche di Base, Cliniche Intensivologiche e Perioperatorie Università Cattolica del Sacro Cuore, Rome Italy; ^3^ Unità Operativa “Medicina di Precisione in Microbiologia Clinica”, Direzione Scientifica Fondazione Policlinico Universitario A. Gemelli IRCCS Rome Italy

**Keywords:** encephalitis, meningitis, PCR assay, syndromic panel

## Abstract

Central nervous system (CNS) infections require prompt and accurate diagnosis to enable timely and targeted antimicrobial therapy. Syndromic PCR‐based assays, such as the QIAstat‐Dx Meningitis/Encephalitis Panel (QIA‐ME), allow rapid detection of key pathogens directly from cerebrospinal fluid (CSF). We conducted a narrative review of seven studies (2023–2025) evaluating the diagnostic performance of QIA‐ME. A total of 1007 clinical CSF samples, ranging from 5 to 585, were retrospectively analyzed, with 8 to 14 targets assessed per study. Positive percent agreement (PPA) ranged from 90.6% to 100%, while negative percent agreement (NPA)—available in only three studies—ranged from 75.0% to 97.7%. In three studies, head‐to‐head comparisons with the BioFire FilmArray Meningitis/Encephalitis Panel (FA‐ME) revealed minimal variation in performance, reinforcing the robustness of QIA‐ME. However, target‐specific limitations were noted, particularly for herpesviruses such as HSV‐1. Methodological heterogeneity across studies—in terms of design, comparator methods, and sample size—limits generalizability. The exclusion of cytomegalovirus and inclusion of *Mycoplasma pneumoniae* and *Streptococcus pyogenes* in the QIA‐ME panel raise concerns about the clinical utility of low‐prevalence targets, especially as *M. pneumoniae* was not detected in any clinical sample. Despite these limitations, the integration of amplification curve analysis and the strong concordance with FA‐ME support QIA‐ME as a valuable tool for CNS infection diagnostics. Prospective real‐world studies are warranted to clarify the clinical value of individual targets and guide appropriate use across varied patient settings.

## Graphical abstract

This review compares the QIAstat‐Dx ME panel with the BioFire FilmArray ME panel across seven studies on CNS infections. The findings support the clinical utility of QIAstat‐Dx, highlighting its diagnostic performance, interpretative advantages, and the need for further real‐world evaluation in high‐risk populations.

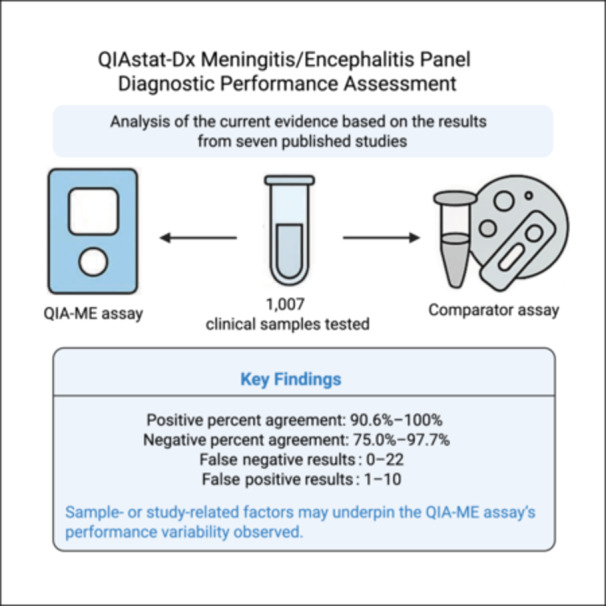

## 1 Introduction

Infectious meningitis and encephalitis are life‐threatening neurological syndromes requiring prompt diagnosis and targeted treatment (Thy et al. 2022; Granerod et al. 2023). A wide range of bacterial, viral, and fungal pathogens may be involved, often with overlapping clinical features. To overcome the limitations of traditional diagnostic workflows (Vetter et al. 2020), syndromic molecular panels have emerged, enabling the simultaneous detection of multiple central nervous system (CNS) pathogens in a single, rapid assay (Couturier and Bard 2019; Dien Bard and McElvania 2020; Olie et al. 2024).

Since its FDA clearance in 2015, the BioFire® FilmArray® Meningitis/Encephalitis Panel (bioMérieux, Marcy l’Étoile, France)—hereafter referred to as FA‐ME—has become widely used in clinical settings. It targets 14 pathogens: six bacteria (*Escherichia coli* K1, *Haemophilus influenzae*, *Listeria monocytogenes*, *Neisseria meningitidis*, *Streptococcus agalactiae*, and *Streptococcus pneumoniae*), seven viruses (cytomegalovirus, enterovirus, herpes simplex virus 1 and 2, human herpesvirus 6, human parechovirus, and varicella‐zoster virus), and one fungus (*Cryptococcus neoformans*/*Cryptococcus gattii*).

In 2022, a second assay—the QIAstat‐Dx Meningitis/Encephalitis Panel (QIAGEN GmbH, Hilden, Germany), hereafter referred to as QIA‐ME—entered the European market. The assay is CE‐IVD marked for in vitro diagnostic use in Europe. This 15‐target assay includes eight bacteria (the same six as FA‐ME, plus *Mycoplasma pneumoniae* and *Streptococcus pyogenes*), six viruses (excluding cytomegalovirus), and *C. neoformans*/*C. gattii*. A key differentiating feature is that QIA‐ME provides semiquantitative data, including amplification curves and threshold cycle (Ct) values. The QIA‐ME assay subsequently received FDA clearance in November 2024, marking its approval for clinical use in the United States, although limited to only nine microbial targets (https://www.accessdata.fda.gov/cdrh_docs/reviews/K242256.pdf).

While both assays aim to improve early diagnosis of CNS infections, uncertainties remain regarding their optimal use in clinical practice (Diallo et al. 2021; Vaugon et al. 2022). Limitations in analytical performance, risks of false positives, and challenges in interpreting certain viral detection results have raised questions about their actual impact (Vetter et al. 2020; Subedi et al. 2025). Compared to FA‐ME, QIA‐ME has been less extensively studied, particularly in real‐world settings.

This review aims to provide a comprehensive assessment of current evidence on the QIA‐ME assay, including study designs, comparator methods, and diagnostic performance metrics, and to explore its emerging role alongside the earlier FA‐ME assay in the diagnostic laboratory.

## 2 Methods

A literature search was conducted to identify relevant studies evaluating the diagnostic performance of the QIAstat‐Dx Meningitis/Encephalitis (QIA‐ME) Panel assay. The following search string was used across Scopus and PubMed databases: (“QIAstat‐Dx” OR “QIAstat Dx” OR “QIAstat”) AND (“Meningitis” OR “Encephalitis”) AND (“Panel” OR “Syndromic test” OR “Molecular diagnostic”). No date restrictions were applied. Only articles published in English and reporting original data on the use of the QIA‐ME assay were included. Conference abstracts, case reports, and review articles were excluded.

This was not a systematic review. Rather, we adopted a narrative approach to allow flexibility in analyzing a heterogeneous body of literature on QIA‐ME performance. Although the initial search strategy employed structured queries in Scopus and PubMed, no formal protocol was preregistered, and PRISMA guidelines were not followed. Studies were selected based on their relevance to the topic and manually screened for eligibility. Full texts were reviewed to extract information on study design, patient populations, comparator methods, and diagnostic performance metrics. The choice of a narrative format reflects the variability across available studies in terms of patient sample number, comparator methods, and pathogen coverage. While this approach facilitates a broader contextual analysis, it may introduce selection bias and limits the reproducibility of the review process. These methodological aspects should be considered when interpreting the findings and the generalizability of the conclusions.

The Scopus search (years 2022–2025) yielded 46 records. Of these, nine articles were considered potentially eligible, along with two related commentaries on the study by Cuesta et al. (2024), one of the articles included in our review. These comprise a published comment and the authors' subsequent reply, both of which will be discussed below. Two articles were excluded: Kyaw et al. (2024) and Şahin et al. (2025). Although both reported results of the QIA‐ME assay on CSF samples analyzed prospectively (Kyaw et al. 2024) or retrospectively (Şahin et al. 2025) to identify infectious causes of suspected meningitis or encephalitis, neither study included a comparative evaluation. Therefore, they were excluded. The PubMed search (years 2022–2025) identified eight records. Of these, 8 were considered potentially eligible. One article—Kyaw et al. (2024), also retrieved in Scopus—was excluded for the same reason as above. Finally, seven articles retrieved from both searches were included in the study.

When not explicitly reported, positive percent agreement (PPA) and negative percent agreement (NPA) were calculated from available data on true positives (TP), false negatives (FN), true negatives (TN), and false positives (FP). Results from the QIA‐ME and/or the FA‐ME assay were considered TP or TN when concordance was observed with the comparator method. Conversely, results from the QIA‐ME and/or FA‐ME assay were classified as FN or FP in cases of discordance, depending on whether the assay yielded a negative or positive result, respectively, that was not confirmed by the comparator method. For consistency, confidence intervals (CIs) for PPA and NPA were calculated using the Wilson score method (SPSS Statistics version 24.0, IBM Corp.). In articles reporting only concordance rates or incomplete data, agreement values were recalculated or estimated based on the available information, as specified in the Table 1 footnotes and described in Section 3.

**Table 1:** Overview of published studies evaluating the QIAstat‐Dx ME Panel assay for the molecular diagnosis of infectious meningitis/encephalitis.

Author name, year	Country	Type (no.) of samples tested^a^	Targets detected across tested samples^b^	Comparator method used per target type			Parameters used for the assay evaluation						
							Positive percent agreement				Negative percent agreement		
				Bacterial	Viral	Fungal	TP/(TP + FN)	%	95% CI		TN/(TN + FP)	%	95% CI
Le Bars et al. 2023	France	CSF (5)	Bacterial: Nme, Spn Viral: HSV‐2, HPeV, VZV	Culture	PCR assays^c^	—	5/5	100	56.5–100		—	—	—
Humisto et al. 2023	Finland	CSF (61)	Bacterial: Hin, Lmo, Nme, Sag, Spn Viral: EV, HHV‐6, HPeV, HSV‐1, HSV‐2, VZV Fungal: Cne/Cga	Culture	PCR assays^d^	Antigen testing and/or microscopy	58/61	95.1	86.5–98.3		—	—	—
Sundelin et al. 2023	Germany, France, Denmark	CSF (585)	Bacterial: Eco, Hin, Lmo, Nme, Sag, Spn Viral: EV, HHV‐6, HPeV, HSV‐1, HSV‐2, VZV Fungal: Cne/Cga	PCR assay^e^	PCR assay^e^	PCR assay^e^	135/146	92.5	87.0–95.7		429/439	97.7	95.9–99.8
Boers et al. 2024	The Netherlands	CSF (106)	Bacterial: Eco, Hin, Lmo, Nme, Sag, Spn, Spy Viral: EV, HHV‐6, HPeV, HSV‐1, HSV‐2, VZV Fungal: Cne/Cga	Culture and/or PCR assays^f^	PCR assays^f^	Antigen testing and/or culture	98/106	92.4	85.8–96.1		—	—	—
Cuesta et al. 2024	Spain	CSF (50)	Bacterial: Lmo, Nme, Sag, Spn Viral: HHV‐6, HSV‐1, HSV‐2, VZV	Culture and/or antigen testing	PCR assays^g^	—	27/28	96.4	82.3–99.4		21/22	95.4	78.2–99.2
Vizcarra et al. 2025	Bolivia	CSF (30)	Bacterial: Eco, Hin, Lmo, Nme, Sag, Spn, Spy Fungal: Cne/Cga	Culture and/or antigen testing, and/or PCR assay^h^	—	Antigen testing and/or microscopy	26/26	100	86.7–100		3/4	75.0	28.8–96.1
Gabrielli et al. 2025	Italy	CSF (170)	Bacterial: Hin, Lmo, Nme, Sag, Spn, Spy Viral: EV, HHV‐6, HPeV, HSV‐1, HSV‐2, VZV Fungal: Cne/Cga	Culture and/or antigen testing	PCR assays^i^	Antigen testing and/or microscopy	154/170	90.6	85.3–94.1		—	—	—

^a^ Of the seven studies summarized, some also included contrived cerebrospinal fluid (CSF) samples—that is, negative CSF samples spiked with a specific microbial pathogen—to expand the range of targets evaluated, as required for the analytical performance assessment of the QIAstat‐Dx Meningitis/Encephalitis (ME) Panel (QIAGEN GmbH, Hilden, Germany), hereafter referred to as QIA/ME. Only clinical CSF samples were considered in this summary to ensure a clinically relevant evaluation of test performance. Data obtained from contrived samples were excluded from the per‐study metrics.

^b^ Include microbial targets for which the QIA/ME assay was evaluated using CSF samples that tested positive in each study. Microbial pathogens detected by the QIA/ME assay (acronyms in parentheses) are listed in alphabetical order as follows: bacterial pathogens—*Escherichia coli* K1 (Eco), *Haemophilus influenzae* (Hin), *Listeria monocytogenes* (Lmo), *Mycoplasma pneumoniae* (Mpn), *Neisseria meningitidis* (Nme), *Streptococcus agalactiae* (Sag), *Streptococcus pneumoniae* (Spn), and *Streptococcus pyogenes* (Spy); viral pathogens—human enterovirus (EV), human herpesvirus 6 (HHV‐6), human parechovirus (HPeV), herpes simplex virus 1 (HSV‐1), herpes simplex virus 2 (HSV‐2), and varicella‐zoster virus (VZV); and fungal pathogens—*Cryptococcus neoformans* (Cne) and *Cryptococcus gattii* (Cga), detected but not differentiated. Target names are provided without indicating the number of tested samples per pathogen. For details on individual microbial pathogens, refer to Table 2.

^c^ Include R‐GENE PCR (bioMérieux, Marcy l’Étoile, France) assays for the individual detection of HPeV and VZV, and the BioGX Viral Meningitis HSV/VZV (Eurobio Scientific, Les Ulis, France) assay, which enables multiplex detection of HSV‐1, HSV‐2, and VZV.

^d^ Include in‐house PCR assays for HHV‐6 and PeV (see study‐specific references), as well as commercial PCR assays. The latter include the Xpert EV (Cepheid, Sunnyvale, CA, USA) assay for EV detection; the artus HSV‐1/HSV‐2 (QIAGEN, Hilden, Germany) assay for HSV‐1/HSV‐2; and the artus VZV (QIAGEN) assay for VZV.

^e^ The Biofire FilmArray ME Panel (bioMérieux) assay, which enables multiplex detection of all QIA/ME targets except *Streptococcus pyogenes* (Spy) and *Mycoplasma pneumoniae* (Mpn), was the only comparator method used across all target types (i.e., bacterial, viral, and fungal) evaluated in the studies.

^f^ Include in‐house PCR assays (i.e., laboratory‐developed tests performed in ISO 15189:2012‐accredited laboratories) for the detection of bacterial and viral pathogens (see study‐specific references). For uncommon bacterial pathogens, a CE‐marked IS‐pro PCR‐based assay was used (see study‐specific reference); this assay profiles bacteria based on species‐specific length variations in the 16S–23S rRNA interspace region.

^g^ Include individual Nanogen Advanced Diagnostics (Palex, Barcelona, Spain) assays for the detection of HHV‐6, HSV‐1/2, and VZV.

^h^ The bioMérieux Biofire FilmArray ME Panel assay (see footnote e for assay details) was used as a comparator method.

^i^ Include the ELITe MGB Kits, a set of singleplex assays used for the quantitative detection of EV, HHV‐6, HSV‐1, HSV‐2, and VZV, and the Meningitis Viral 2 ELITe MGB Panel assay, a multiplex assay used for the qualitative detection of HPeV. All assays are manufactured by ELITechGroup (Torino, Italy).

Additional symbols and abbreviations used in the table: CI, confidence interval; TN, true negative; TP, true positive; –, not applicable or not calculable (i.e., used when the target was not evaluated or when no CSF samples testing negative were included in a given study).

While concordance generally refers to the overall agreement between two methods, PPA and NPA provide a more granular assessment of assay performance, specifically in positive and negative cases. In this review, PPA and NPA were prioritized to ensure a standardized and clinically meaningful summary of assay performance. However, in two of the articles included in this review (Cuesta et al. 2024; Vizcarra et al. 2025), concordance was also formally assessed using Cohen's kappa coefficient, which adjusts for agreement expected by chance. In contrast, our comparative analysis focused on recalculated PPA and NPA values wherever possible, to ensure consistent interpretability across studies.

## 3 Results

### 3.1 Studies Evaluating the QIA‐ME Assay: Design and Comparators

The literature search identified seven studies published between 2023 and 2025 (Le Bars et al. 2023; Humisto et al. 2023; Sundelin et al. 2023; Boers et al. 2024; Cuesta et al. 2024; Vizcarra et al. 2025; Gabrielli et al. 2025), following the introduction of the QIA‐ME assay into the European market. These studies, all reporting original data on the use of the QIA‐ME assay, are summarized in Table 1 and listed according to their chronological publication dates: March 2023, April 2023, September 2023, January 2024, April 2024, January 2025, and April 2025, respectively.

All included studies retrospectively evaluated archived CSF samples that had been previously aliquoted and stored frozen until QIA‐ME testing. These samples were collected from patients with clinical suspicion of meningitis or encephalitis and had already undergone diagnostic workup using routine laboratory methods. Accordingly, most samples had been classified as positive—or, in some studies, negative—based on laboratory‐developed tests, commercial molecular assays (e.g., PCR‐based assays), or conventional microbiological methods (e.g., antigen testing)—together defined as standard of care (SoC)—used as reference methods. In certain studies, the comparator method consisted solely of the FA‐ME panel, while others employed a combination of FA‐ME with culture and/or other molecular assays (see footnotes to Table 1 for details).

When a direct head‐to‐head comparison between QIA‐ME and FA‐ME was made, the results are reported separately in Section 3.4. This distinction clarifies the QIA‐ME assay's diagnostic performance relative to the SoC and identifies studies with formal comparative evaluations.

### 3.2 Diagnostic Performance of the QIA‐ME Assay Across All Pathogens Evaluated

A total of 1007 clinical CSF samples were analyzed across the seven studies included in this review. The number of samples tested per study ranged from 5 in the pilot evaluation by Le Bars et al. (2023) to 585 in the multicenter study by Sundelin et al. (2023), which currently represents the most extensive assessment of the QIA‐ME assay.

The number of pathogen targets evaluated varied across studies, depending on the availability of positive samples for each target. Between 8 and 14 targets were assessed per study, typically covering common bacterial and viral pathogens, with five studies also including fungal pathogens.

PPA values were reported in all studies, ranging from 90.6% to 100%, depending on the pathogens and comparator methods employed. These methods included culture, antigen testing, microscopy, and PCR‐based assays, either alone or in combination. In contrast, NPA values were available in only three studies, ranging from 75.0% to 97.7%.

FP results were reported in four of the seven studies. Sundelin et al. (2023), comparing QIA‐ME to FA‐ME, identified 10 FP results: 2 each for *H. influenzae* (Hin), human herpesvirus 6 (HHV‐6), herpes simplex virus 2 (HSV‐2), and varicella‐zoster virus (VZV), plus 1 each for human enterovirus (EV) and *S. pneumoniae* (Spn). However, using SoC results, 5 of these 10 cases were reclassified as TP results: 2 HSV‐2, 1 HHV‐6, 1 EV, and 1 Spn. Boers et al. (2024), using SoC as comparator, reported 1 FP result for VZV in a sample that was TP for *S. pyogenes* (Spy), with corresponding Ct values of 35.9 (VZV) and 20.9 (Spy). Similarly, Cuesta et al. (2024) found 1 FP result involving VZV (Ct 38.5), consistent with a prior diagnosis of VZV encephalitis made 15 days earlier. A single FP result was also reported in the study by Vizcarra et al. (2025), where Hin was detected by QIA‐ME (Ct 30.6 in a 1:3 diluted CSF sample), but not by culture or FA‐ME. Notably, Hin was isolated from blood cultures collected around the same time, and the patient responded to ceftriaxone‐targeted therapy. This case suggests that QIA‐ME may have detected a true CNS infection not captured by the comparator methods.

Table 1 summarizes the sample sizes, pathogens tested, comparator methods, and diagnostic performance metrics of the QIA‐ME assay across all studies included.

### 3.3 Diagnostic Performance of the QIA‐ME Assay by Single Pathogens Evaluated

FN results were reported in five of the seven studies included. No FN results were observed in the studies by Le Bars et al. (2023) and Vizcarra et al. (2025).

Among bacterial pathogens, *S. pneumoniae* (Spn) was not detected in 2 of 17 positive samples (11.8%) in the study by Sundelin et al. (2023), and in 1 of 11 positive samples (9.1%) in the study by Boers et al. (2024). Additional FN results were reported for *H. influenzae* (Hin) and *L. monocytogenes* (Lmo): 1 of 5 positive samples (20.0%) for Hin in Sundelin et al. (2023), and 1 of 7 positive samples (14.3%) for Lmo in Boers et al. (2024).

Among viral pathogens, FN results were mainly observed in the studies by Humisto et al. (2023), Sundelin et al. (2023), and Gabrielli et al. (2025), with only one FN result reported by Cuesta et al. (2024). Human herpesvirus 6 (HHV‐6) and herpes simplex virus 1 (HSV‐1) showed the highest number of FN results, with 9 each: 1 in Humisto et al. (2023), 1 in Sundelin et al. (2023), and 7 in Gabrielli et al. (2025) for HHV‐6; and 1 in Humisto et al. (2023), 1 in Cuesta et al. (2024), and 7 in Gabrielli et al. (2025) for HSV‐1. These were followed by varicella‐zoster virus (VZV) with 7 FN results—3 in Sundelin et al. (2023) and 4 in Gabrielli et al. (2025); human enterovirus (EV) with 6 FN results—2 in Sundelin et al. (2023) and 4 in Gabrielli et al. (2025); and herpes simplex virus 2 (HSV‐2) with 3 FN results—1 in Humisto et al. (2023) and 2 in Sundelin et al. (2023). In the study by Sundelin et al. (2023), using SoC results, 4 of the 11 FN results were reclassified as TN results: 2 Spn, 1 Hin, and 1 EV.

Regarding *C. neoformans*/*C. gattii* (Cne/Cga), FN results were reported only by Boers et al. (2024), with six cases.

A detailed summary of FN results by pathogen and study is provided in Table 2.

**Table 2:** Positive detection results of the QIAstat‐Dx ME Panel assay stratified by pathogen type in cerebrospinal fluid (CSF) samples across published studies.

Type of pathogen (acronym)	No. of true positives/(true positives + false negatives)^a^						
	Le Bars, 2023	Humisto, 2023	Sundelin, 2023	Boers, 2024	Cuesta, 2024	Vizcarra, 2025	Gabrielli, 2025
*Bacterial pathogen*							
*E. coli* K1 (Eco)	—	—	1/1	1/1	—	3/3	—
*H. influenzae* (Hin)	—	2/2^b^	**4/5**	7/7	—	2/2	1/1
*L. monocytogenes* (Lmo)	—	5/5	1/1	**6/7**	3/3	5/5	2/2
*N. meningitidis* (Nme)	1/1	1/1	1/1	10/10	1/1	5/5	3/3
*S. agalactiae* (Sag)		6/6	3/3	1/1	2/2	1/1	2/2
*S. pneumoniae* (Spn)	1/1	6/6	**15/17**	**10/11**	5/5	7/7	9/9
*S. pyogenes* (Spy)	—	—	—	3/3	—	1/1	1/1
*Viral pathogen*							
Enterovirus (EV)	—	6/6	**7/9**	24/24	1/1	—	**24/28**
Human herpesvirus 6 (HHV‐6)	—	**1/2**	**9/10**	3/3	2/2	—	**30/37**
Human parechovirus (HPeV)	1/1	4/4	—	5/5	—	—	2/2
Herpes simplex virus 1 (HSV‐1)	—	**9/10**	20/20	6/6	**6/7**	—	**29/36**
Herpes simplex virus 2 (HSV‐2)	1/1	**11/12**	**21/23**	4/4	4/4	—	15/15
Varicella‐zoster virus (VZV)	1/1	6/6	**52/55**	11/11	3/3	—	**36/40**
*Fungal pathogen*							
*C. neoformans*/*C. gattii* (Cne/Cga)	—	1/1	1/1	**7/13**	—	2/2	2/2
Total pathogens tested	5/5	58/61	135/146	98/106	27/28	26/26	156/178^c^

*Note:* Bold indicates targets with at least one false‐negative result, while – indicates not applicable (i.e., the target was not evaluated in that study).

^a^ Samples in each study were tested with the QIA‐ME assay retrospectively, and results were assessed as true positive or false negative by comparison with the reference method results (see Table 1 footnotes and text for comparator methods used). False positive results are not reported in this table; see Table 1 for NPA values when available.

^b^ One additional positive sample yielded an invalid (but not negative) result with the QIA‐ME assay and was therefore excluded from the denominator.

^c^ Of the 178 results, 20 were bacterial‐positive results (from 20 CSF samples), while the remaining 158 were viral‐positive results (from 150 CSF samples). The 158 positive results included 28 for EV, 37 for HHV‐6, 2 for HPeV, 36 for HSV‐1, 15 for HSV‐2, and 40 for VZV. Eleven samples tested positive for two pathogens: 8 with dual viral targets and 3 with viral plus bacterial or fungal targets. The 22 false‐negative results corresponded to 16 samples positive for one viral pathogen, 5 for two viral pathogens, and 1 for viral and fungal pathogens.

### 3.4 QIA‐ME Versus FA‐ME: A Head‐to‐Head Comparison Analysis

In three of the seven studies included in this review, QIA‐ME and FA‐ME assays were directly compared by testing the same set of CSF samples, which had been pre‐classified as positive for specific pathogens based on reference methods independently defined in each study. Overall, a substantial concordance was observed between the two assays across all evaluated targets. While no difference was reported in the study by Le Bars et al. (2023), in the study by Humisto et al. (2023), one *S. pneumoniae* (Spn) false‐negative (FN) result occurred with the FA‐ME assay, whereas QIA‐ME correctly detected the pathogen. Similarly, in the study by Cuesta et al. (2024), FA‐ME failed to detect Spn in one sample that tested positive with QIA‐ME and showed three FN results for herpes simplex virus 1 (HSV‐1), while QIA‐ME reported only one FN result for the same target, which occurred in the same sample as one of the FA‐ME FN results.

While Table 3 summarizes the number of TP and FN results for each assay in these head‐to‐head comparisons, a detailed analysis of results from the study by Cuesta et al. (2024) revealed additional targets detected by the FA‐ME assay in several samples that were not captured by QIA‐ME. This occurred in three samples that tested concordantly positive with both assays, in which FA‐ME additionally detected the following pathogens: (i) HSV‐1, herpes simplex virus 2 (HSV‐2), *H. influenzae* (Hin), and Spn in one sample that was positive for *S. agalactiae* (Sag); (ii) Sag in one sample that was positive for HSV‐2; and (iii) HSV‐1 in one sample that was positive for varicella‐zoster virus (VZV).

**Table 3:** Pathogen‐positive detection results obtained by QIAstat‐Dx ME Panel (QIA‐ME) and FilmArray Meningitis/Encephalitis Panel (FA‐ME) assays in selected studies.

Type of pathogen (acronym)	No. of true positives/(true positives + false negatives)^a^					
	Le Bars, 2023		Humisto, 2023		Cuesta, 2024	
	QIA‐ME	FA‐ME	QIA‐ME	FA‐ME	QIA‐ME	FA‐ME
*Bacterial pathogen*						
*E. coli* K1 (Eco)	—	—	—	—	—	—
*H. influenzae* (Hin)	—	—	2/2^b^	3/3^c^	—	—
*L. monocytogenes* (Lmo)	—	—	5/5	5/5	3/3	3/3
*N. meningitidis* (Nme)	1/1	1/1	1/1	1/1	1/1	1/1
*S. agalactiae* (Sag)			6/6	6/6	2/2	2/2
*S. pneumoniae* (Spn)	1/1	1/1	6/6	**5/6**	5/5	**3/4** d
*Viral pathogen*						
Enterovirus (EV)	—	—	6/6	6/6	1/1	1/1
Human herpesvirus 6 (HHV‐6)	—	—	**1/2**	**1/2**	2/2	2/2
Human parechovirus (HPeV)	1/1	1/1	4/4	4/4	—	—
Herpes simplex virus 1 (HSV‐1)	—	—	**9/10**	**9/10**	**6/7**	**4/7**
Herpes simplex virus 2 (HSV‐2)	1/1	1/1	**11/12**	**11/12**	4/4	4/4
Varicella‐zoster virus (VZV)	1/1	1/1	6/6	6/6	3/3	3/3
*Fungal pathogen*						
*C. neoformans*/*C. gattii* (Cne/Cga)	—	—	1/1	1/1	—	—
Total pathogens tested	5/5	5/5	58/61	58/62	27/28	23/27

*Note:* Bold indicates targets with at least one false‐negative result, while – indicates not applicable (i.e., the target was not evaluated in that study).

^a^ Samples in each study were tested with both the QIA‐ME and FA‐ME assays, and results were assessed as true positive or false negative by comparison with the reference method results (see Table 1 footnotes and text for comparator methods used). False positive results are not reported in this table; see Table 1 for NPA values when available.

^b^ One additional positive sample yielded an invalid (but not negative) result with the QIA‐ME assay and was therefore excluded from the denominator.

^c^ The sample that yielded an invalid result with the QIA‐ME assay (see footnote b) tested positive with the FA‐ME assay and was therefore included in the denominator.

^d^ One additional positive sample yielded an invalid (but not negative) result with the FA‐ME assay and was therefore excluded from the denominator.

In addition, nine samples yielded FP results with FA‐ME: seven were positive for HSV‐1, while two showed multiple pathogens. Specifically, one of these two samples was positive for Hin, Spn, and cytomegalovirus, and the other for *E. coli* K1 and Sag.

## 4 Discussion

### 4.1 Diagnostic Performance of the QIA‐ME Assay and Inter‐Study Variability

Our analysis of the available literature on the diagnostic performance of the QIA‐ME panel—a newer syndromic molecular assay compared to the earlier FA‐ME—highlights key findings that may inform its role in the meningitis/encephalitis diagnostic workflow. The QIA‐ME panel includes 15 microbial targets (excluding cytomegalovirus) and generally showed PPA and NPA values above 90%, indicating excellent performance. An exception was the study by Vizcarra et al. (2025), where NPA was substantially lower. The number of false‐negative (FN) and false‐positive (FP) results varied considerably across studies, ranging from 0 to 22 FNs and 1 to 10 FPs. Gabrielli et al. (2025) and Sundelin et al. (2023) reported the highest FN and FP counts, respectively. Notably, Boers et al. (2024) reported FN results for fungal pathogens (6 out of 13 positives), and this was the only study with a sufficiently large sample size for *C. neoformans*. These findings suggest that study‐specific or sample‐related factors may contribute to the variability in QIA‐ME performance.

A possible explanation for the difference in performance metrics across studies lies in the heterogeneity of study sample sizes. For instance, Vizcarra et al. (2025) reported an NPA of 75.0% based on only three TNs out of four total negative CSF samples, which is clearly a limited data set. In contrast, Sundelin et al. (2023) reported a much higher NPA of 97.7%, calculated from 439 CSF samples including 10 FPs. This comparison highlights how small sample sizes can skew performance estimates, reinforcing the need to interpret PPA and NPA values in the context of the data set's scale.

Importantly, the QIA‐ME assay received FDA 510(k) clearance following a multicenter evaluation that included both United States and European sites. It is plausible that the European component of this evaluation corresponded to the centers included in Sundelin et al. (2023). Further supporting the robustness of that study, Sundelin et al. also performed a discordant result analysis that led to the reclassification of nearly half of the initial FP and FN results, thereby improving the reliability of their estimates.

Another source of inter‐study variability is the type of comparator method used. For example, Gabrielli et al. (2025) reported as many as 22 FNs for viral targets. That study excluded CSF samples that tested negative for all pathogens and relied on singleplex PCR assays rather than multiplex PCR assays as the reference standard. Such differences in comparator assay format and analytical sensitivity may have influenced the number of FN results observed.

### 4.2 Head‐to‐Head Comparisons of QIA‐ME With the FA‐ME Assay

Additional support for the diagnostic reliability of the QIA‐ME assay comes from studies that conducted head‐to‐head comparisons with the FA‐ME assay, in which both assays were applied to the same set of CSF samples and evaluated against a common reference method. The limited variation in performance metrics between the two assays across these studies reinforces the reliability of QIA‐ME. For this reason, we focused on the subset of studies (*n* = 3; see Table 3, Section 3.4 of the Results) that included such comparisons (Le Bars et al. 2023; Humisto et al. 2023; Cuesta et al. 2024). Among them, the study by Cuesta et al. (2024) was further discussed in a commentary by Paranhos‐Baccalà et al. (2024), who advocated for the continued relevance of the FA‐ME assay. Interestingly, their argument drew on data from Sundelin et al. (2023), one of the most comprehensive QIA‐ME/FA‐ME performance evaluations to date, which actually demonstrated strong overall agreement between QIA‐ME and FA‐ME. This observation, rather than weakening the role of QIA‐ME, highlights its diagnostic reliability and consistency across different settings and comparators. It thus supports its adoption as a reliable alternative to the FA‐ME assay in routine clinical practice.

A closer look at pathogen‐specific performance highlights how certain viral targets, such as HHV‐6 or HSV‐1, were more frequently associated with false‐negative (FN) results. HSV‐1 yielded FNs in three of the included studies: Humisto et al. (2023), Cuesta et al. (2024), and Gabrielli et al. (2025). In Humisto and Cuesta—both of which conducted head‐to‐head comparisons—FN results for HSV‐1 showed complete concordance between assays, with 9/10 FNs shared in Humisto and 6/7 versus 4/7 missed by QIA‐ME and FA‐ME, respectively, in Cuesta. These findings suggest that HSV‐1 detection may be a shared diagnostic challenge. By contrast, Sundelin et al. (2023) reported perfect detection of HSV‐1 (20/20 cases), further highlighting inter‐study variability and the possible influence of sample characteristics or methodological differences.

As noted in the commentary by Paranhos‐Baccalà et al. (2024), the higher HSV‐1 positivity rate observed with the QIA‐ME assay compared to the FA‐ME assay in the study by Cuesta et al. (2024) may reflect a nonrepresentative cohort—characterized by a large proportion of immunosuppressed patients, frequent CSF samples with normal or minimally altered biochemistry, and possible HSV‐1 reactivation or contamination from traumatic lumbar punctures—all factors that may increase the risk of false‐positive results. In their reply, Cuesta et al. (2024a) speculated that the ability to visualize the amplification curve and its corresponding Ct value with the QIA‐ME assay might help mitigate this risk and improve result interpretation, particularly in borderline cases. They emphasized that this visualization goes beyond the Ct value alone and includes the shape of the amplification curve, which can provide important clues to the reliability of signal detection.

While the commentary by Paranhos‐Baccalà et al. (2024) noted that a similar functionality is now available for the FA‐ME assay through the newly introduced BioFire FIREWORKS application, the broader discussion underscores how the integration of semi‐quantitative or kinetic amplification data may enhance the clinical contextualization of molecular results (Candel et al. 2024). In particular, access to *C*_t_ values and amplification curves allows clinical microbiologists to differentiate between true infections and potential contamination or latent viral shedding, especially when interpreting low‐level positive results. This added granularity may be especially helpful in immunocompromised patients or in cases where pathogens such as HHV‐6 are detected, for which clinical significance can be ambiguous (Vetter et al. 2020; Smit et al. 2025). Integrating such data into the clinical workflow supports more informed diagnostic decisions and could mitigate the risks of overtreatment or misinterpretation, particularly in the context of low‐prevalence targets or unexpected findings (Candel et al. 2024; Subedi et al. 2025).

### 4.3 Clinical Relevance of Low‐Prevalence Targets in the QIA‐ME Assay

The clinical utility of any multiplex PCR assay depends largely on the epidemiological relevance of its targets. From this perspective, the decision to remove cytomegalovirus (CMV) from the QIA‐ME assay and introduce *M. pneumoniae* (Mpn) and *S. pyogenes* (Spy) deserves careful consideration (Thomson and Bertram 2001; Razonable et al. 2020). Ideally, such modifications should be guided by real‐world diagnostic needs rather than by marketing‐driven differentiation from competing assays. Notably, through the clinical studies reviewed, none reported positive detections for Mpn (see Tables 1 and 2). Sundelin et al. (2023) attempted to address this by including a large number of contrived samples (data not shown) to assess the QIA‐ME assay's analytical performance for low‐prevalence targets. As for Spy, it was detected in only three studies—Boers et al. (3/3 positives), Vizcarra et al. and Gabrielli et al. (1/1 positive each)—within study cohorts of 106, 30, and 170 CSF samples, respectively.

These findings raise important questions about the value of including low‐prevalence pathogens in routine syndromic testing. While Mpn and Spy may be relevant in specific settings—such as pediatric populations or during outbreaks—their inclusion in broad‐range panels requires strong justification. In this regard, Humisto et al. (2023) acknowledged that although their study did not test for Mpn or Spy, broader use of rapid molecular assays might help uncover pathogens in otherwise undiagnosed cases. However, this comes with potential risks, particularly the possibility of increased false‐positive results.

Cross‐reactivity is a known issue that can reduce specificity. For example, *Cutibacterium acnes* or *Mycoplasma genitalium* might yield false‐positive Mpn signals, while *Haemophilus haemolyticus* could mimic *Haemophilus influenzae*. Additionally, the detection of certain viruses (e.g., HHV‐6) may reflect latent or noncausal infections rather than active disease. As noted by Humisto et al. (2023), these issues highlight the importance of cautious, context‐aware interpretation of results—especially for rare targets with uncertain clinical significance. A prospective, real‐world evaluation of QIA‐ME performance in both general and high‐risk populations (e.g., patients with HIV or transplant recipients) would be valuable to better define the diagnostic impact of these uncommon targets and guide their integration into clinical workflows.

### 4.4 Clinical Interpretability and Potential Impact of the QIA‐ME Assay

The included studies were highly heterogeneous in terms of patient sample size, comparator methods, and pathogen coverage. This heterogeneity limits comparability across studies and affects the generalizability of the findings. Despite these limitations, some shared elements support a reasoned reflection on the potential clinical implications of implementing the QIA‐ME assay.

The multiplex format may particularly benefit patients at high risk for rapid clinical deterioration or with broad differential diagnoses, such as neonates, immunocompromised individuals, or patients in intensive care settings (Candel et al. 2024; Smit et al. 2025). In these populations, a fast and broad diagnostic approach may support earlier pathogen detection and inform prompt treatment decisions.

However, the risk of false‐negative or false‐positive results must be carefully weighed. Missed diagnoses may lead to delayed or inappropriate treatment, whereas unexpected detections—especially of low‐prevalence or low‐pathogenicity targets—can result in unnecessary therapies. The interpretation of such findings should be contextualized by clinical microbiologists, considering amplification curve characteristics, local epidemiology, and clinical plausibility.

In this context, the availability of Ct values and amplification curves, as provided by the QIA‐ME assay, may offer a key advantage for experienced laboratories. These data allow more nuanced interpretation of borderline or clinically ambiguous results, potentially supporting antimicrobial stewardship efforts (Subedi et al. 2025).

While the added value of a negative result is clear for common pathogens—helping to rule out treatable infections and avoid unnecessary antibiotics—its clinical interpretation becomes more uncertain when negative findings lead to treatment de‐escalation in the absence of other supporting data (Vetter et al. 2020; Smit et al. 2025). Many pathogens that can cause CNS infections are not currently covered by multiplex assays (Olie et al. 2024), which further limits their standalone use.

Therefore, the real‐world clinical utility of QIA‐ME should be assessed through prospective studies in specific care settings such as emergency departments, intensive care units, and resource‐limited environments (Candel et al. 2024; Milburn et al. 2024). In these contexts, rapid and user‐friendly assays may facilitate early diagnosis, although the actual impact on antimicrobial use or clinical outcomes may be limited. For instance, over 20 prospective studies have evaluated the clinical utility of FA‐ME, often demonstrating benefits like faster time to targeted therapy or shorter stay in the intensive care unit—but these advantages were mostly linked to positive results (Smit et al. 2025). As noted by Vetter et al. (2020), current evidence does not support multiplex PCR assays (e.g., FA‐ME) as standalone tools for routine clinical care. Accordingly, the clinical value of QIA‐ME—including its potential to reduce inappropriate treatment or unnecessary hospitalization—remains to be demonstrated through similar prospective studies.

### 4.5 Concluding Remarks

This narrative review has intrinsic limitations. We reanalyzed the testing results study by study, focusing exclusively on clinical CSF samples, which were used in all included studies. However, differences in study design, inclusion criteria, and comparator methods limit the direct comparability of the findings. In some studies, only SoC‐positive samples were tested using multiplex PCR assays (QIA‐ME, FA‐ME, or others), introducing potential selection bias. Additionally, the definitions of true and false results varied, with reference methods ranging from singleplex to other multiplex PCR assays or even clinical adjudication. Nonetheless, we aimed to highlight consistent trends by prioritizing data from head‐to‐head comparisons and studies with clearly reported results from clinical samples.

Despite the limited number of available studies, our analysis of clinical CSF samples supports the use of the QIA‐ME assay as a diagnostic tool for meningitis/encephalitis. Its performance appears comparable to that of the FA‐ME assay, although target‐specific limitations persist. Further real‐world data are warranted to guide optimal assay selection and the clinical interpretation of results.

## Author Contributions

**Flora Marzia Liotti:** conceptualization (lead), writing – original draft (supporting), formal analysis (lead), writing – review and editing (equal). **Brunella Posteraro:** conceptualization (lead), writing – original draft (lead), formal analysis (supporting), writing – review and editing (equal). **Maurizio Sanguinetti:** conceptualization (supporting), writing – review and editing (equal). **Giulia De Angelis:** conceptualization (supporting), writing – original draft (supporting), formal analysis (supporting), writing – review and editing (equal).

## Ethics Statement

The authors have nothing to report.

## Conflicts of Interest

Some authors have received honoraria from bioMérieux (BioFire Diagnostics). The authors declare no conflicts of interest.
